# THSD1 Is a Multifaceted Regulator in Health and Disease

**DOI:** 10.3390/biomedicines13061292

**Published:** 2025-05-24

**Authors:** Mengjun Dai, Kuizhi Qu, Sophie Liu, Zhen Xu, Yan-Ning Rui

**Affiliations:** Department of Neurosurgery, McGovern Medical School, The University of Texas Health Science Center at Houston, Houston, TX 77030, USA

**Keywords:** THSD1, vascular integrity, developmental disorder, cancer, autophagy, intracranial aneurysm

## Abstract

Thrombospondin Type 1 Domain-Containing Protein 1 (THSD1) is a transmembrane protein increasingly recognized for its critical roles in vascular biology and disease pathogenesis. Initially identified as a marker of hematopoietic stem and endothelial cells during embryogenesis, THSD1 has since been implicated in a wide spectrum of physiological and pathological processes. This paper consolidates current knowledge on THSD1, with a focus on its roles in vascular integrity, perinatal disorders, and tumorigenesis. In vascular systems, THSD1 promotes focal adhesion assembly and suppresses autophagy-mediated adhesion turnover, thereby stabilizing endothelial attachment and maintaining barrier function. Genetic and functional studies support its protective role against intracranial aneurysms and hemorrhagic vascular disorders. THSD1 mutations have also been linked to perinatal disorders such as nonimmune hydrops fetalis and congenital vascular anomalies, suggesting a broader role in embryonic vascular patterning. Moreover, emerging evidence indicates that THSD1 acts as a tumor and metastasis suppressor, with potential anti-angiogenic properties, although its role in cancer remains to be fully defined. This paper not only consolidates existing knowledge but also identifies critical research gaps, providing a robust foundation for future investigations into the biology and clinical relevance of THSD1.

## 1. Introduction

Thrombospondin Type 1 Domain-Containing Protein 1 (THSD1) is a single-span transmembrane protein that has garnered increasing attention since its initial identification as a novel cell surface molecule expressed in hematopoietic stem cells and endothelial cells during murine embryogenesis embryogenesis [1]. Structurally, THSD1 features an extracellular thrombospondin type 1 repeat domain, which mediates interactions with extracellular matrix components such as CD36, thereby facilitating endothelial cell adhesion and modulating angiogenic signaling. It also contains a transmembrane helix and an intracellular domain suggestive of signaling capabilities. Through an in silico database search, THSD1 was originally named Tmtsp (transmembrane molecule with thrombospondin module) and recognized for its potential as a marker of primitive hematopoietic and endothelial lineages. Over the past two decades, there has been an increasing number of studies positioning THSD1 as a critical regulator in vascular disease pathogenesis and endothelial regulation.

This paper aims to summarize the current understanding of THSD1 across three major areas: “THSD1 in vascular integrity and intracranial aneurysms”, “THSD1 in developmental and perinatal disorders”, and “THSD1 in cancer and emerging roles”. By integrating genetic, functional, and mechanistic insights, we want to elucidate the contribution of THSD1 to endothelial biology, its pathological implications, and its therapeutic potential. From its initial discovery as a hematopoietic stem cell and endothelial marker to its current status as a multifaceted regulator, THSD1 offers a compelling narrative of how a single protein can influence diverse physiological and disease states. In summary, this paper highlights key areas of progress while also pointing toward unresolved questions and future directions in THSD1 research. The flowchart of this review is presented in Supplemental Figure S1.

## 2. THSD1 in Vascular Integrity and Intracranial Aneurysms

The most extensively studied role of THSD1 is in maintaining vascular integrity, particularly in the context of intracranial aneurysm (IA) and subarachnoid hemorrhage, conditions characterized by weakening and rupture of the cerebral artery wall [2]. Foundational evidence was provided by Santiago-Sim et al., who identified a nonsense mutation (R450X) in THSD1 that perfectly segregated with IA in a large family [3] (summarized in Supplemental Table S1). This nonsense mutation introduces a premature stop codon, which is predicted to cause loss of function of the THSD1 protein through truncation. Given THSD1’s role in stabilizing focal adhesions and maintaining endothelial cell anchorage to the basement membrane, such loss of function is believed to compromise endothelial integrity, making the vessel wall more susceptible to hemodynamic stress and rupture.

Further analysis of 507 unrelated IA probands revealed additional rare variants (e.g., L5F, R460W, E466G) in 1.6% of sporadic cases—a frequency significantly enriched compared to 89,040 control chromosomes [3]. Interestingly, intracranial hemorrhage has been observed in two THSD1-deficient vertebrate models—zebrafish and mice—with the hemorrhage localized to the subarachnoid space in the latter. Additional studies from other groups further support the role of THSD1 in IA pathogenesis. Chen et al. identified two single-nucleotide polymorphisms (SNPs), rs3803264 and rs1054000, in the THSD1 gene that are significantly associated with increased risk of hemorrhagic stroke in a Chinese Han population. Both SNPs were correlated with altered mRNA expression levels of THSD1 [4]. Notably, IA-linked THSD1 variants have been identified not only in the Chinese Han population but also in Northwestern European, African American, and Hispanic populations, highlighting ethnic diversity in genetic predisposition [3,5]. Collectively, these findings suggest that both coding mutations and expression-level changes that reduce THSD1 activity may act through similar loss-of-function mechanisms, weakening endothelial stability and promoting IA formation and rupture.

A mechanistic understanding of the role of THSD1 in vascular biology and disease has progressively deepened over time. Although the focus of this review is on THSD1, it is worth noting that its key functional domain—the thrombospondin type 1 repeat (TSR)—shares evolutionary and structural homology with thrombospondin-1 (TSP-1), a prototypical extracellular matrix protein. TSP-1 is known to be rapidly released from platelet α-granules upon thrombin stimulation and acts as a matricellular modulator in wound healing, angiogenesis, and immune regulation [6]. It also transiently integrates into the extracellular matrix during tissue development and repair, highlighting the dynamic roles of TSR-containing proteins in both homeostatic and pathological settings. These properties suggest that the TSR domain of THSD1 may confer regulatory functions that extend beyond structural adhesion, including vascular remodeling and response to injury.

Functional studies of THSD1 knockdown in human umbilical vein endothelial cells (HUVECs) using siRNA for two days impaired focal adhesion (FA) stability, as evidenced by reduced FA number and size and decreased cell adhesion to collagen I-coated plates [3]. FA stability is a critical determinant of endothelial barrier function and vascular integrity. Rui et al. demonstrated that THSD1 interacts with FA proteins and stabilizes endothelial attachment to the basement membrane [7]. Furthermore, Xu et al. revealed that THSD1 negatively regulates autophagy, thereby suppressing autophagy-mediated FA turnover and enhancing FA stability [8].

Beclin 1 is a core regulator of autophagy initiation and plays a central role in nu-cleating autophagosome formation. It functions by forming a multiprotein complex with ATG14, VPS34, and VPS15—collectively known as the class III PI3K complex, which is specifically involved in autophagosome biogenesis [9]. The interaction between Beclin 1 and ATG14 is essential for targeting VPS34 activity to pre-autophagosomal membranes, leading to the production of PI3P, a lipid crucial for autophagosome nucleation. Disruption of this interaction impairs early autophagic signaling and vesicle formation.

Through detailed signaling analysis, the Beclin 1 pathway was identified as critical for THSD1-mediated FA stability. Under normal physiological conditions, THSD1 interacts with FAK and promotes FAK-mediated phosphorylation of Beclin 1 at tyrosine 233. This post-translational modification inhibits Beclin 1’s ability to bind ATG14, thereby preventing the assembly of the VPS34 complex at autophagic initiation sites and suppressing autophagosome formation. Conversely, when THSD1 is absent or downregulated, reduced FAK activity leads to dephosphorylation of Beclin 1 at Y233, which in turn restores its interaction with ATG14 and triggers autophagy initiation. As a result, Beclin 1 binds to ATG14 and activates autophagosome biogenesis, leading to the degradation of FAs in endothelial cells [8]. It is worth noting that autophagy-mediated FA turnover (FA-phagy) is a selective process [10]; therefore, THSD1-mediated FA-phagy likely involves specific cargo receptors or adaptors that have yet to be identified. This autophagic regulation highlights a dynamic interplay between THSD1 and cellular degradation pathways, potentially amplifying its protective role. The disruption of focal adhesion integrity and enhancement of FA-phagy in the setting of THSD1 deficiency ultimately lead to compromised endothelial cohesion and increased vascular permeability. Mechanistically, loss of THSD1 weakens the anchorage of endothelial cells to the basement membrane and promotes cytoskeletal disorganization, resulting in widened intercellular junctions and impaired barrier function. In vivo, this manifests as intracranial hemorrhage in zebrafish and mouse models, and it may contribute to lesion-prone sites in human vasculature.

The influence of THSD1 extends beyond intracranial aneurysms to other vascular contexts. In atherosclerosis, THSD1 expression is downregulated by pro-atherogenic factors such as TNFα and upregulated by the anti-atherogenic cytokine IL-10, suggesting a dynamic regulatory role in atherosclerosis prevention [11]. Furthermore, in an ApoE knockout mouse model of advanced atherosclerosis, THSD1 overexpression reduces intraplaque hemorrhage by 45%, accompanied by decreased macrophage infiltration and necrotic core size [11]. Additionally, Ranasinghe et al. associated THSD1 variants with spontaneous coronary artery dissection [12]. A summary of key studies investigating THSD1 in vascular biology and disease is provided in Table 1. The cellular basis of vascular protective function of THSD1 lies in its endothelial specificity and interactions with focal adhesions. Several groups confirmed high THSD1 expression in endothelial cells, with minimal expression in smooth muscle cells, consistent with its role in maintaining endothelial barrier function [1,3].

## 3. THSD1 in Developmental and Perinatal Disorders

The role of THSD1 in early development is rooted in its expression during embryogenesis. Takayanagi et al. demonstrated that THSD1 is highly expressed in the yolk sac and the aorta–gonad–mesonephros regions, which mark the onset of definitive hematopoiesis [1]. In THSD1 knockout mice, early-stage cerebral hemorrhage was observed, indicating vascular instability during developmental or perinatal stages [3]. These findings are further supported by zebrafish models, where thsd1 knockdown induces intracranial hemorrhages in embryos 2–3 days post-fertilization, highlighting a potential role for THSD1 in developmental vascular disorders [3,11].

THSD1 has also been implicated in nonimmune hydrops fetalis (NIHF), a serious perinatal disorder characterized by abnormal fluid accumulation in the fetus or newborn. Shamseldin et al. identified two homozygous THSD1 mutations (C206Y and R224X) in two Saudi families with NIHF [13], while Abdelrahman et al. reported a novel homozygous eight-nucleotide deletion (c.1163-1170delGGCCAGCC) in four Emirati siblings with NIHF, accompanied by congenital cardiac defects and hemangiomas [14]. In addition, Al Rawi et al. described a case of an extremely premature infant harboring a homozygous THSD1 variant (Q521X), further expanding the phenotypic spectrum associated with THSD1 deficiency [15,16].

Beyond the genetic association, recent clinical analyses suggest that THSD1 deficiency may disrupt fetal vascular integrity and lymphatic development, potentially contributing to fluid imbalance in nonimmune hydrops fetalis [17]. In affected neonates harboring THSD1 mutations, postmortem findings often reveal generalized edema, ascites, and pleural effusions [15]—hallmarks of impaired vascular or lymphatic drainage. While the exact mechanisms remain elusive, it is hypothesized that endothelial dysfunction due to THSD1 loss increases capillary leakage and impairs vessel maturation during embryogenesis [11]. Given THSD1’s role in focal adhesion and autophagy regulation in endothelial cells [8], similar pathways may underline the pathophysiology of nonimmune hydrops fetalis. In this regard, THSD1 could serve as a potential diagnostic marker or therapeutic target in select cases of unexplained hydrops, especially when accompanied by vascular malformations or hemangiomas [14].

Although these clinical observations strongly implicate THSD1 in developmental and perinatal disorders, the underlying molecular mechanisms remain incompletely understood. It is plausible that increased vascular permeability contributes to these pathological outcomes, especially in the context of THSD1 deficiency. Whether THSD1-mediated regulation of autophagy and focal adhesion turnover plays a mechanistic role in these conditions remains to be determined. Further experimental studies are required to dissect these pathways and clarify how THSD1 dysfunction contributes to developmental anomalies and perinatal complications.

## 4. THSD1 in Cancer and Emerging Roles

Emerging evidence suggests that THSD1 plays a role in tumorigenesis across multiple cancer types. THSD1 mutations have been identified in gastric cancer and are associated with unfavorable survival outcomes [18]. Large-scale proteomic studies by two independent groups have confirmed that THSD1 serves as a robust biomarker for gastric cancer [19,20]. In colorectal cancer and esophageal squamous cell carcinoma cell lines, THSD1 expression is significantly downregulated, potentially through promoter methylation [21,22]. Similarly, THSD1 promoter methylation has been observed in lung adenocarcinoma and correlates significantly with reduced overall survival [23]. In addition to epigenetic regulation, THSD1 expression can be inducible by external stressors. THSD1 was found to be upregulated in human primary fibroblasts following radiation exposure, as well as in lung cancer cells treated with phenanthriplatin [24,25]. Overexpression of THSD1 in esophageal squamous cell carcinoma cell lines inhibits colony formation, suggesting a tumor-suppressive function [22]. In addition, genetic duplication of THSD1 was associated with improved survival outcomes in sporadic childhood cancers [26].

Some studies have directly assessed the clinical relevance of THSD1 in cancer cohorts. In gastric adenocarcinoma, Jin et al. [19] conducted a proteome-wide Mendelian randomization and single-cell transcriptomic analysis using tumor specimens and linked THSD1 expression to poor overall survival and increased peritoneal metastasis rates. Another study using monochromosome transfer and microarray analysis demonstrated that transfection of wild-type THSD1 into SLMT-1 cells significantly reduced colony-forming ability, providing functional evidence for its tumor-suppressive activity [22]. These functional insights, together with clinical expression data, strengthen the case for THSD1 as a diagnostic and prognostic biomarker in human cancers.

Recent mechanistic studies further elucidate THSD1’s role as a tumor suppressor and therapeutic target. In addition to its regulatory effects on focal adhesions, THSD1 appears to modulate the tumor microenvironment by influencing vascular stability and immune cell infiltration [8]. Its loss could accelerate progression in conjunction with other oncogenic stressors [7]. This includes enhanced tumor angiogenesis, increased focal adhesion turnover, and cytoskeletal remodeling, all of which facilitate metastasis [19]. While THSD1-targeting strategies are not yet clinically available, these findings highlight their potential in precision oncology. Collectively, these observations suggest that THSD1 may serve as a diagnostic and prognostic biomarker across multiple cancer types and may function as a tumor and metastasis suppressor, although the underlying molecular mechanisms remain to be fully elucidated.

Angiogenesis is a critical process in cancer progression. The presence of a thrombospondin type 1 domain in THSD1 suggests potential anti-angiogenic properties [27]. While THSD1 does not directly affect neovascular growth in zebrafish or murine retina models [1,11], its role in maintaining vascular integrity may indirectly influence tumor vasculature and microenvironment. Since THSD1 suppresses autophagy-mediated focal adhesion turnover, this mechanism may also be exploited by cancer cells for migration and metastasis [8]. Nonetheless, the definitive role of THSD1 in cancer biology remains to be elucidated. Further studies are needed to dissect its molecular mechanisms and evaluate its potential as a therapeutic target. These findings position THSD1 as a versatile regulator with promising applications in cancer diagnosis, prognosis, and therapy.

An emerging role for THSD1 has also been identified in the field of extracellular vesicle (EV) biology, particularly in the context of physiological stress and systemic adaptation. Recent studies investigating EV profiles in military personnel undergoing operational stress and exercise-induced challenges have highlighted THSD1 as a specific marker of large extracellular vesicles, particularly apoptotic bodies, the largest EV subtype derived from dying or stressed cells. Across several human cohort studies, THSD1 consistently appeared as a surface marker enriched in large EV populations, including those isolated during intense military training, resistance exercise, and stress-induced physiological responses [28,29,30,31]. These findings suggest that THSD1 may be a distinguishing feature of EVs associated with cellular turnover or apoptotic signaling, offering potential utility in EV-based biomarker discovery. Although the functional role of THSD1 in EV biogenesis or trafficking remains unclear, its consistent presence on apoptotic bodies implies a broader involvement in intercellular communication under stress or damage conditions. This emerging link not only broadens the biological relevance of THSD1 but also opens new avenues to explore its utility as a biomarker of physiological stress and systemic resilience, further extending its diagnostic potential beyond traditional vascular and oncologic contexts.

## 5. Conclusions/Discussion

THSD1 has emerged as a multifaceted regulator of vascular biology with significant implications across diverse physiological and pathological processes. From its early identification as a marker of hematopoietic and endothelial lineages to its recently uncovered roles in vascular integrity, perinatal disorders, and cancer, THSD1 represents a critical molecular node linking endothelial stability, focal adhesion dynamics, and autophagy regulation.

Focal adhesions are dynamic protein complexes that anchor cells to the extracellular matrix and mediate intracellular signaling. Impairment of focal adhesion disrupts cell adhesion, migration, and survival, contributing to a range of pathological processes. In vascular systems, defective focal adhesions compromise endothelial integrity, leading to increased permeability and hemorrhage. In cancer, deregulated focal adhesion turnover facilitates invasion and metastasis by enhancing cellular motility. Thus, precise regulation of focal adhesion is critical for maintaining tissue homeostasis and preventing disease.

**Figure 1:**
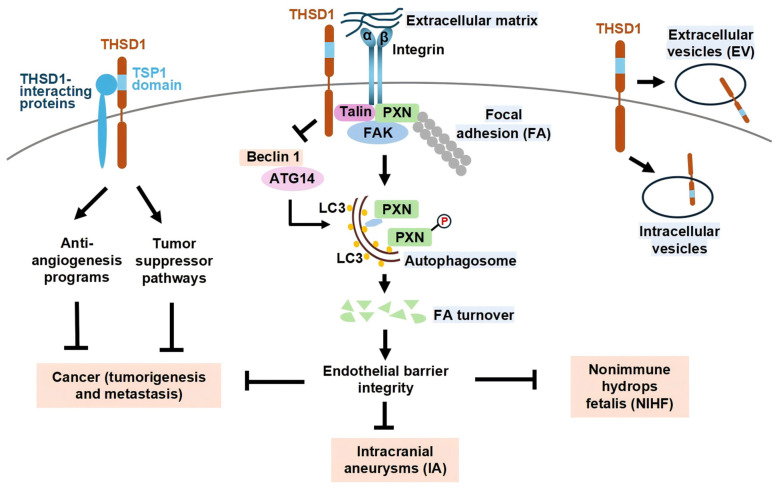
**Role of THSD1 in health and disease.** Focal adhesions (FAs) are simplified here as protein complexes consisting of integrin α and β subunits that connect the extracellular matrix to intracellular proteins such as talin, paxillin (PXN), and focal adhesion kinase (FAK). THSD1 directly interacts with these FA-associated proteins. Under physiological conditions, THSD1 prevents Beclin 1 from binding to ATG14, thereby suppressing Beclin 1-dependent autophagy and focal adhesion turnover (FA-phagy). This mechanism stabilizes focal adhesions, maintaining endothelial barrier integrity and protecting against the development of intracranial aneurysms. Beyond vascular integrity, THSD1 may interact with other currently unidentified proteins, potentially through its thrombospondin type 1 (TSP1) domain. These uncharacterized interactions could function as suppressors of tumorigenesis or metastasis through various intracellular signaling pathways. The molecular mechanisms linking THSD1 to anti-angiogenesis and tumor suppressor programs, as well as its potential involvement in cancer and nonimmune hydrops fetalis (NIHF), remain largely unknown. Alternatively, THSD1-mediated endothelial barrier stabilization may indirectly influence cancer progression or perinatal disorders, such as nonimmune hydrops fetalis (NIHF). Intriguingly, THSD1 is also present within intracellular vesicles, such as endosomes, and extracellular vesicles, including apoptotic bodies released from cells, though the functional role of THSD1 within these extracellular vesicles remains to be determined.

Current evidence strongly supports its protective role in preventing intracranial aneurysms, its involvement in perinatal conditions such as nonimmune hydrops fetalis, and its potential tumor-suppressive and anti-metastatic functions in various cancers (Figure 1). Despite growing interest and supporting data, the mechanistic underpinnings of THSD1 function in these conditions remain incompletely understood. Further studies are warranted to validate its utility as a diagnostic biomarker and to explore its potential as a therapeutic target.

While much of the current understanding of THSD1 stems from global knockout mouse models, the phenotypes observed in these models reflect the systemic loss of THSD1, making it difficult to isolate tissue-specific contributions. To better elucidate the endothelial-specific roles of THSD1, particularly in vascular integrity and disease, endothelial cell-specific knockout models would be more informative. Such models would allow for a more precise dissection of its functions in the vascular endothelium without the confounding effects of systemic loss.

For disease-related studies, knock-in mouse models harboring disease-relevant THSD1 mutations, such as the R450X variant associated with intracranial aneurysms or the C206Y mutation identified in NIHF, would offer invaluable tools to evaluate causal relationships between THSD1 dysfunction and disease development. These models would enable researchers to determine whether such mutations are sufficient to induce aneurysms, NIHF, or other vascular anomalies in vivo. Importantly, these mice could serve as robust preclinical platforms to uncover downstream molecular mechanisms and evaluate potential interventions targeting THSD1 pathways.

Interestingly, to date, no cancer or tumor incidence has been reported in THSD1 knockout mice, raising the possibility that THSD1 loss alone may not be sufficient to initiate tumorigenesis. However, additional oncogenic stress or genetic alterations, such as crossing with cancer-prone mouse strains, may be necessary to uncover its role in cancer progression. It is plausible that loss of THSD1 may accelerate tumor development, potentially through mechanisms such as enhanced tumor angiogenesis or increased focal adhesion turnover, which are known to facilitate cancer cell invasion and metastasis. Exploring these possibilities could reveal THSD1 as a novel regulator of the tumor microenvironment and a promising therapeutic target in oncology.

It is also important to note that many existing studies on THSD1 in cancer have been based on bulk transcriptomic analyses, which limit the ability to distinguish cell-type-specific contributions of THSD1 to tumor initiation and progression. For instance, downregulation of endothelial THSD1 could increase vascular permeability, promoting tumor cell dissemination and microenvironmental remodeling. Conversely, loss of THSD1 expression in epithelial cells may act as a cell-autonomous oncogenic stressor contributing directly to tumorigenesis. Emerging technologies such as single-cell and spatial transcriptomics offer a promising avenue to overcome these limitations and may reveal cell-specific THSD1-associated signaling networks across various cancer types, advancing our understanding of its context-dependent roles and enhancing its potential as a precision biomarker and therapeutic target.

These mechanistic insights also reinforce the potential of THSD1 as a clinically useful biomarker. Biomarkers play a central role in disease diagnosis, prognosis, and therapeutic stratification by offering measurable indicators of pathological processes or treatment response. In vascular and oncologic disorders, molecular biomarkers help identify high-risk individuals, monitor disease progression, and guide personalized therapy. In this context, THSD1 has emerged as a promising biomarker candidate owing to its endothelial-specific expression and mechanistic involvement in focal adhesion stability, autophagy regulation, and vascular integrity. Clinical studies have reported downregulation or mutation of THSD1 in intracranial aneurysms, perinatal disorders, and multiple cancers, supporting its potential relevance in diagnostic and prognostic frameworks. Nonetheless, important challenges remain. THSD1 expression is relatively low in some adult tissues, potentially limiting its detection in noninvasive biosamples. To fully realize the clinical utility of THSD1, further large-scale validation studies and integration into multiplex biomarker panels will be necessary.

### Future Directions

In the future, to further elucidate the role of THSD1 in health and disease, further studies should develop refined in vivo models, such as knock in mice carrying disease-relevant mutations or crossed with cancer-prone strains, to assess causal relationships in tumorigenesis and vascular pathology. Emerging techniques like single-cell and spatial transcriptomics will also be essential to resolve cell-type specific functions of THSD1 in complex tissues. Clinically, large-scale patient-based studies are needed to validate THSD1 as a biomarker and explore its therapeutic potential, especially in the context of multiplex biomarker panels and microenvironment-targeted interventions.

## Figures and Tables

**Table 1:** Summary of key studies investigating THSD1 in vascular biology and disease.

Study	Disease/Context	Experimental Model/Method	Key Findings	Quantitative Data
Santiago-Sim et al. (2016) [3]	IA and SAH	Genetic sequencing of 507 IA probands	THSD1 mutation is a potential genetic risk factor for IA/SAH	Variant frequency: 1.6% in IA probands vs. 0 in 89,040 controls
Rui et al. (2017) [7]	Focal adhesions	siRNA knockdown in HUVECs	THSD1 stabilizes focal adhesions via interaction with FA proteins	FA area decreased; endothelial detachment increased
Chen et al. (2023) [4]	Hemorrhagic stroke	Case-control study in Chinese Han population	SNP rs3803264 and abnormal THSD1 mRNA associated with stroke	Allele frequency OR > 1.5; *p* < 0.05
Xu et al. (2024) [8]	Vascular autophagy	HUVECs and mouse models	THSD1 suppresses Beclin 1-mediated autophagy of focal adhesions	Beclin1 Y233 phosphorylation decreased after THSD1 loss
Sauvigny et al. (2020) [5]	IA	Exome sequencing (n = 38)	THSD1 variants detected in IA+SAH patients	Multiple variants with CADD > 20
Haasdijk et al. (2016) [11]	Atherosclerosis	Mouse model with THSD1 overexpression	THSD1 preserves vascular integrity and reduces intraplaque hemorrhage	Intraplaque hemorrhage reduced by 45% in THSD1-OE mice
Ranasinghe et al. (2023) [12]	Spontaneous coronary artery dissection (SCAD) with leukoencephalopathy	Case report: single patient with rare THSD1 mutation	Rare THSD1 variant may be associated with SCAD and neurological phenotype	1 patient with SCAD and white matter changes + THSD1 mutation

## Supplementary Materials

The following supporting information can be downloaded at: https://www.mdpi.com/article/10.3390/biomedicines13061292/s1, Figure S1. The flow diagram of study selection. Table S1. THSD1 variants associated with Familial IA.

## Abbreviations

The following abbreviations are used in this manuscript:

EV	Extracellular vesicle
FA	Focal adhesion
IA	Intracranial aneurysm
NIHF	Nonimmune hydrops fetalis
THSD1	Thrombospondin Type 1 Domain-Containing Protein 1
