# Supplementary material for: THSD1 Is a Multifaceted Regulator in Health and Disease
Source: Biomedicines. 2025 May 24;13(6):1292. doi: 10.3390/biomedicines13061292 (PMC12189160; doi:10.3390/biomedicines13061292)
Supplement: Supplementary file 1 [file biomedicines-13-01292-s001.zip › biomedicines-3578304-supplementary.pdf]

Figure S1. The flow diagram of study selection

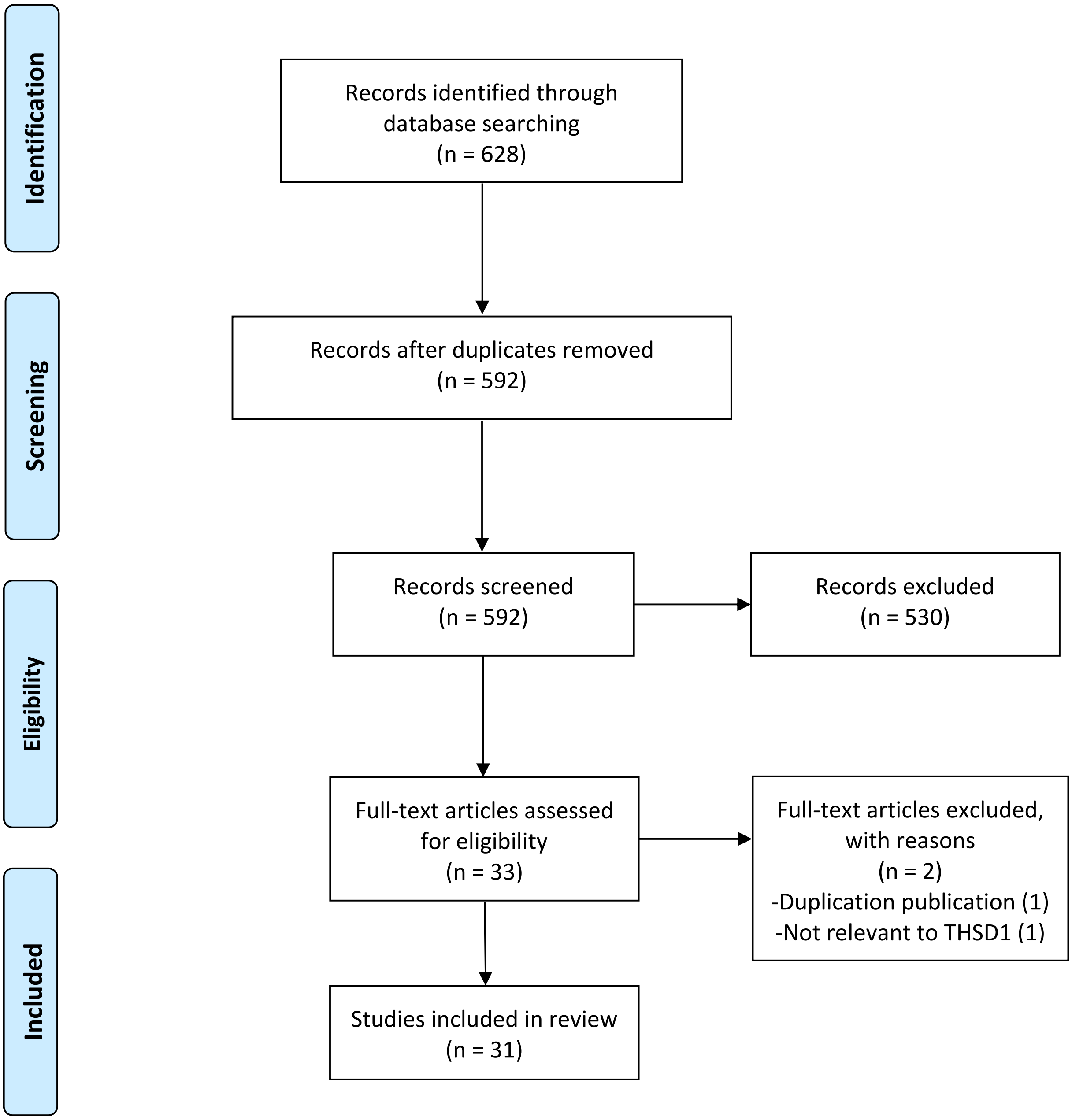

Table S1. THSD1 variants associated with Familial IA

Variant (cDNA/protein)	Variant Type	Familial/Sporadic	Notes on Association	Reference
c.1348C>T / p.R450X	Nonsense	Familial (9 affected members)	Clear segregation with familial IA	Santiago-Sim et al., 2016 [1]
c.15G>C / p.L5F	Missense	Sporadic	Found in sporadic IA patients	
c.1378C>T / p.R460W	Missense	Sporadic	Found in sporadic IA patients	
c.1397A>G / p.E466G	Missense	Familial (2 affected members)	Observed in familial IA cases	
c.1799G>A / p.G600E	Missense	Sporadic	Found in sporadic IA patients	
c.1916C>T / p.P639L	Missense	Sporadic	Found in sporadic IA patients	
c.1958C>T / p.T653I	Missense	Sporadic	Found in sporadic IA patients	
c.2323T>C / p.S775P	Missense	Sporadic (African American patient)	Found in sporadic IA patients;	
c.1666G>C / p.Gln556Glu	Missense	Familial	Found in one IA patient with positive family history	Sauvigny et al., 2020 [2]
c.871C>T / p.Glu291Lys	Missense	Familial	Found in four IA patient with positive family history in two of them	
c.592G>C / p.Gln198Glu	Missense	Familial	Found in one IA patient with positive family history	

1. Santiago-Sim, T.; Fang, X.; Hennessy, M.L.; Nalbach, S.V.; DePalma, S.R.; Lee, M.S.; Greenway, S.C.; McDonough, B.; Hergenroeder, G.W.; Patek, K.J.; et al. THSD1 (Thrombospondin Type 1 Domain Containing Protein 1) Mutation in the Pathogenesis of Intracranial Aneurysm and Subarachnoid Hemorrhage. *Stroke* **2016**, *47*, 3005-3013, doi:10.1161/STROKEAHA.116.014161.
2. Sauvigny, T.; Alawi, M.; Krause, L.; Renner, S.; Spohn, M.; Busch, A.; Kolbe, V.; Altmuller, J.; Loscher, B.S.; Franke, A.; et al. Exome sequencing in 38 patients with intracranial aneurysms and subarachnoid hemorrhage. *J Neurol* **2020**, *267*, 2533-2545, doi:10.1007/s00415-020-09865-6.
